# Nopaline-type Ti plasmid of *Agrobacterium* encodes a VirF-like functional F-box protein

**DOI:** 10.1038/srep16610

**Published:** 2015-11-20

**Authors:** Benoît Lacroix, Vitaly Citovsky

**Affiliations:** 1 Stony Brook University, Department of Biochemistry and Cell Biology, Stony Brook, NY 11794-5215, USA

## Abstract

During *Agrobacterium*-mediated genetic transformation of plants, several bacterial virulence (Vir) proteins are translocated into the host cell to facilitate infection. One of the most important of such translocated factors is VirF, an F-box protein produced by octopine strains of *Agrobacterium*, which presumably facilitates proteasomal uncoating of the invading T-DNA from its associated proteins. The presence of VirF also is thought to be involved in differences in host specificity between octopine and nopaline strains of *Agrobacterium*, with the current dogma being that no functional VirF is encoded by nopaline strains. Here, we show that a protein with homology to octopine VirF is encoded by the Ti plasmid of the nopaline C58 strain of *Agrobacterium*. This protein, C58VirF, possesses the hallmarks of functional F-box proteins: it contains an active F-box domain and specifically interacts, via its F-box domain, with SKP1-like (ASK) protein components of the plant ubiquitin/proteasome system. Thus, our data suggest that nopaline strains of *Agrobacterium* have evolved to encode a functional F-box protein VirF.

Members of the *Agrobacterium* genus are phytopathogenic bacteria with the unique ability to transfer and integrate a segment of their own DNA (T-DNA) into the genome of their plant hosts^1^,^2^. The genes naturally transferred are expressed in the transformed plant cells and induce uncontrolled cell division (*e*.*g*., crown galls in the case of *Agrobacterium tumefaciens*) and production of opines, small molecules providing a source of carbon and nitrogen for the bacteria^3^. The transfer and integration of *Agrobacterium* T-DNA relies on a set of proteins encoded by bacterial *vir* (virulence) genes located on a specialized Ti (tumor inducing)-plasmid, and on interaction of these Vir proteins with host cell factors^1^,^2^. Besides the *vir* genes absolutely required for transformation, several non-essential *vir* genes are conserved in Ti-plasmids of different *Agrobacterium* strains. They likely represent host range factors required for efficient transformation of specific hosts, or provide a competitive advantage in the complex rhizosphere environment where several strains may be competing for the same host. *vir*F, one of these non-essential genes, was originally described as responsible for difference of virulence between two main *A. tumefaciens* strains: octopine and nopaline (named after the nature of the opines produced in tumors) on several specific hosts. Indeed, a nopaline-specific *Agrobacterium* strain (C58) was only weakly virulent on *Nicotiana glauca*, as opposed to strains (*e.g*., A6) harboring the octopine-type Ti-plasmid; the difference in virulence was found to depend on the *vir*F locus of the octopine-specific *Agrobacterium* strains^4^. Expression of octopine-type *vir*F resulted in efficient transformation of *N. glauca* by the C58 strain, whether *vir*F was expressed in bacteria^4^ or in the host plant^5^, suggesting that VirF activity was effected in the plant cells. It was thus concluded that the C58 strain either contained no *vir*F locus at all or its *vir*F was not functional. Subsequently, it was found that octopine-type VirF is translocated into the host cell^6^ and represents an F-box protein which functions as a subunit of the SCF (Skp1-Cul1-F-box protein) ubiquitin E3 ligase complex^7^,^8^,^9^,^10^ in the host cell. Indeed, octopine-type VirF interacts with ASK proteins, the plant equivalents of yeast Skp1^11^, and triggers proteasomal degradation of the plant protein VIP1 involved in the transformation process^12^. Thus, VirF, the first F-box protein identified in prokaryotes^11^ represents a bacterial pathogen effector that interferes with the host ubiquitin/proteasome system (UPS)^13^.

That such an important virulence function as an F-box protein is not conserved between major *Agrobacterium* strains does not make biological sense. Indeed, the Ti-plasmid from C58 *Agrobacterium* strain contains in its *vir* region a gene—*Atu6154*, which we term here C58*vir*F—whose protein product C58VirF shares homology with the octopine-type VirF. A *vir*F locus was also found in several Ti-plasmids from *Agrobacterium vitis*, suggesting that the presence of *vir*F homologs is widespread in different Agrobacterium species and strains^14^. Here, we investigated the function of C58VirF and demonstrated its specific interaction with the plant UPS machinery, which suggests its functionality as a true F-box protein. Potentially, the level of virulence of octopine and nopaline strains of *Agrobacterium* on different hosts depends, at least in part, on specificity of their VirF F-box proteins.

## Results

### Amino acid sequence analysis of C58VirF

**Figure 1:**
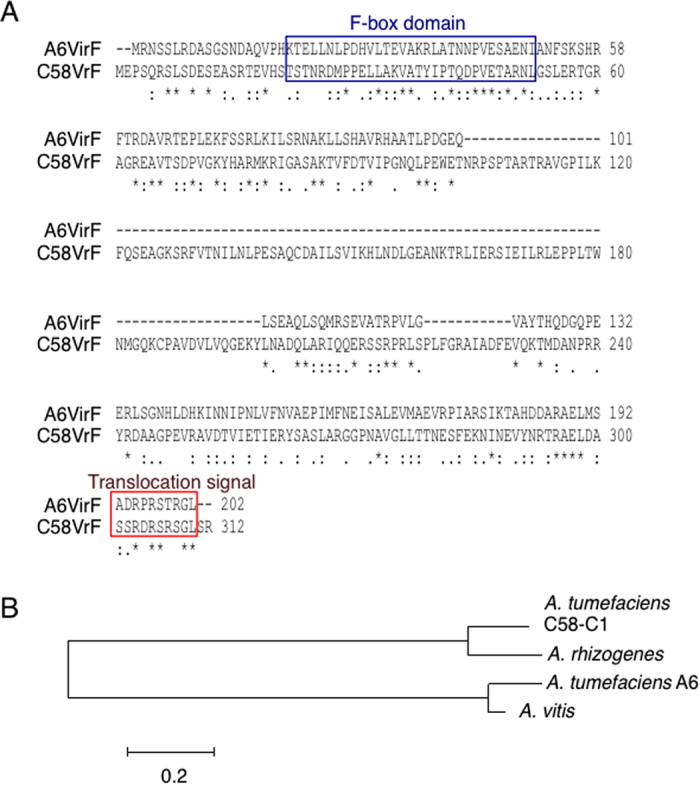
Sequence analyses of C58VirF. (**A**) Alignment of *A. tumefaciens* VirF protein sequences from the octopine-specific A6 strain (A6VirF, GenBank accession number AF24281.1) and the nopaline-specific C58-C1 strain (C58VirF, GenBank accession number AE007871.2) was performed by ClustalW2 (ver. 2) at EMBL-EBI (http://www.ebi.ac.uk/Tools/msa/clustalw2/) using the default settings. Symbols designations: “*” identical residues, “:” conserved substitutions, “.” semi-conserved substitutions. The conserved F-box domain and T4SS export signal are delineated by blue and red boxes, respectively. (**B**) Phylogenetic tree of the VirF protein orthologs from *A. tumefaciens* C58-C1, *A. tumefaciens* A6, *A. vitis* S4 and *A. rhizogenes* was constructed using the Molecular Evolutionary Genetics Analysis (MEGA, version 6.0.5 for Mac OS) tool (http://www.megasoftware.net). Bar = 0.2 amino acid substitutions per site.

The C58*vir*F gene is located in the *vir* region of the Ti-plasmid of the *Agrobacterium* C58-C1 strain, between *vir*H and the region containing the *vir*A-E loci. By comparison with octopine-type VirF from the A6 strain (A6VirF), the C58*vir*F-encoded protein, C58VirF, is noticeably longer*, i.e.*, 312 amino acid residues versus 202 residues, respectively (Fig. 1A). Homology between these two proteins is observed in an 85-residue-long N-terminal region and in the 100-residue-long C-terminal region whereas the central region of the C58VirF protein, about 100 amino acid-long as well, is absent in the octopine-type ortholog. Whereas the ProfileScan software did not detect any functional domains in the C58VirF sequence, manual analysis of sequence alignment revealed a region of homology, corresponding to the octopine-type F-box domain, including some of the most conserved amino acid residues of the F-box domains^15^,^16^. In addition, a strong homology is found in the C-terminus of the protein, which corresponds to the arginine-rich bacterium-to-host cell translocation signal; this signal allows a Vir protein to be recognized as substrate by the bacterial type IV secretion system (T4SS), which then transports it into the host cytoplasm^17^. Indeed, C58VirF has been shown to be transferred from *Agrobacterium* to plant cell^17^.

A phylogenetic tree constructed with VirF protein sequences from the two major *Agrobacterium* strains—nopaline-specific *A. tumefaciens* C58 and octopine-specific *A. tumefaciens* A6— as well as from two other *Agrobacterium* species, *A. vitis* S4, and *A. rhizogenes*—revealed two distinct groups (Fig. 1B): one containing *A. tumefaciens* C58 and *A. rhizogenes*, and the other containing *A. tumefaciens* A6 and *A. vitis*. Thus, C58VirF and A6VirF, apart from the homology found in the regions corresponding to their F-box and translocation signal domains, are evolutionary distant from each other. One of the hallmarks of most *Agrobacterium vir* genes is their inducibility by plant secondary metabolites, such as acetosyringone^18^,^19^. The C58*vir*F locus indeed contains a conserved regulatory *vir* box element in its promoter region (data not shown). A study of another nopaline-specific *Agrobacterium* strain, SAKURA, which is almost identical to C58 in its *vir* region sequence, showed that expression of SAKURA*vir*F is induced by acetosyringone^20^. Most likely, therefore, C58*vir*F also represents a true *vir*-type gene, albeit belonging to a group different from that of the classical VirF protein, A6VirF.

### Subcellular localization in plant cells

**Figure 2:**
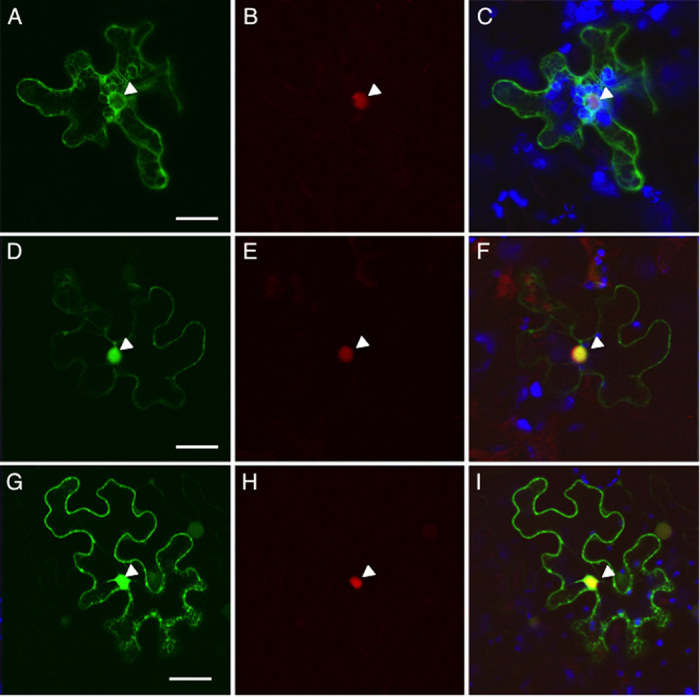
Subcellular localization of C58VirF and A6VirF in plant cells. The indicated combinations of fluorescently-tagged proteins were transiently expressed in *N.* *benthamiana* leaf mesophyll cells. (**A–C**) GFP-GUS-C58VirF + RFP-NLS. (**D–F**) GFP-C58VirF + RFP-NLS. (**G–I**) GFP-GFP-A6VirF + RFP-NLS. GFP fluorescence is in green, RFP fluorescence is in red, overlapping GFP and RFP fluorescence is in yellow, plastid autofluorescence is in blue. Location of the cell nucleus is indicated by a white arrowhead. Images are single confocal sections. Bars = 20 μm.

Previous studies suggested that C58VirF is transferred from *Agrobacterium* to plant via the T4SS^17^, which delivers the exported bacterial proteins into the recipient cell, first into the cytoplasm and then to a specific compartment in which the protein functions. The specific localization of C58VirF in the host cell, however, remained unknown. Thus, we tagged C58VirF with a GFP-GUS tag, which is a fusion between green fluorescent protein (GFP) and β-glucuronidase (GUS); GFP-GUS, due to its relatively large size would preclude non-specific diffusion of the relatively small C58VirF into the cell nucleus. GFP-GUS-C58VirF was then coexpressed with RFP-NLS, an NLS-containing red fluorescent protein (RFP) that served as internal reference marker for the nuclear compartment. Fig. 2A,C shows that expression of GFP-GUS-C58VirF resulted in GFP fluorescence localized overwhelmingly in the cell cytoplasm and in a perinuclear region. As expected, the RFP-NLS marker accumulated almost exclusively in the cell nucleus, and it did not colocalize with coexpressed GFP-GUS-C58VirF (Fig. 2B,C). These results indicate that C58VirF does not possess active nuclear localization signals (NLSs). Consistently, subcellular localization prediction software PSORT (http://psort.hgc.jp) detected no known specific subcellular localization signals in C58VirF. However, C58VirF is a small protein, and its molecular mass of ca. 34.5 kDa is within the 40–60 kDa size exclusion limit of the nuclear pore^21^. Thus, passive entry of at least some fraction of the intracellular pool of C58VirF into the nucleus cannot be excluded. Indeed, when tagged with a single GFP molecule, C58VirF was found both in the cytoplasm and in the nucleus of the plant cell (Fig. 2D,F), with the nuclear population of GFP-C58VirF colocalizing with RFP-NLS (Fig. 2E,F).

Surprisingly, although octopine VirF-VIP1 complexes are known to accumulate in the cell nucleus^12^, subcellular localization of the octopine VirF itself has not been examined. We transiently expressed octopine VirF fused to a GFP tandem tag (GFP-GFP); similarly to GFP-GUS-C58VirF, the combined molecular mass of GFP-GFP-A6VirF is above the size exclusion limit of the nuclear pore^21^. GFP-GFP-A6VirF was nucleocytoplasmic (Fig. 2G,I). The nuclear portion of GFP-GFP-A6VirF colocalized with coexpressed RFP-NLS, which was entirely nuclear (Fig. 2H,I). These data suggest that VirF is present both in the cytoplasm and the nucleus of the host cell during infection by the octopine-type *Agrobacterium*.

### Interaction with the ASK components of the plant SCF complex

**Figure 3:**
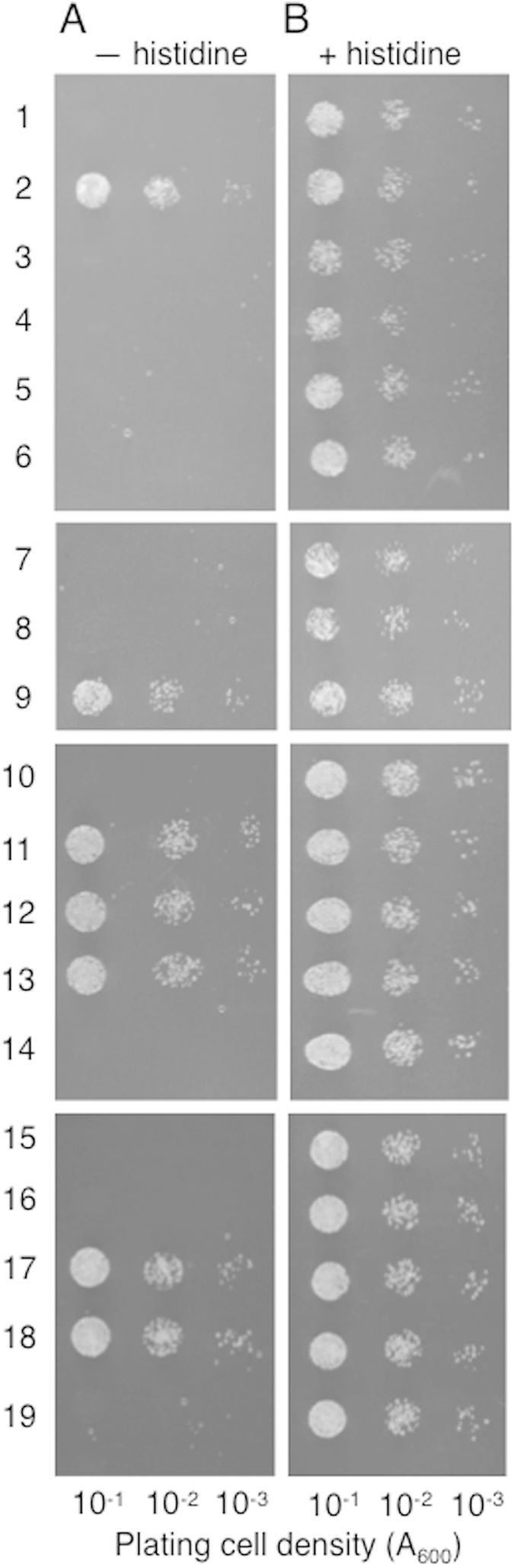
Specific interaction between C58VirF and ASK proteins in the yeast two-hybrid system. (**A**) Cell growth in the absence of histidine, tryptophan and leucine. (**B**) Cell growth in the absence of tryptophan and leucine. Lane 1, LexA-C58VirF + Gal4AD; lane 2, LexA-C58VirF + Gal4AD-ASK1, lane 3, LexA-C58VirF + Gal4AD-VirE3; lane 4, LexA-C58VirF + Gal4AD-VirD2; lane 5, LexA-C58VirF + Gal4AD-VIP1; lane 6, LexA-C58VirF + Gal4AD-VIP2; lane 7, LexA-VirE2 + Gal4AD-C58VirF; lane 8, LexA-VirE2 + Gal4AD-VirD2; lane 9, LexA-VirE2 + Gal4AD-VIP1; lane 10, LexA-C58VirF + Gal4AD; lane 11, LexA-C58VirF + Gal4AD-ASK1; lane 12, LexA-C58VirF + Gal4AD-ASK2; lane 13, LexA-C58VirF + Gal4AD-ASK4; lane 14, LexA-C58VirF + Gal4AD-ASK5; lane 15, LexA-C58VirF + Gal4AD-ASK10; lane 16, LexA-C58VirF + Gal4AD-ASK11; lane 17, LexA-C58VirF + Gal4AD-ASK13; lane 18, LexA-C58VirF + Gal4AD-ASK18; lane 19, LexA-C58VirF + Gal4AD-ASK21.

**Figure 4:**
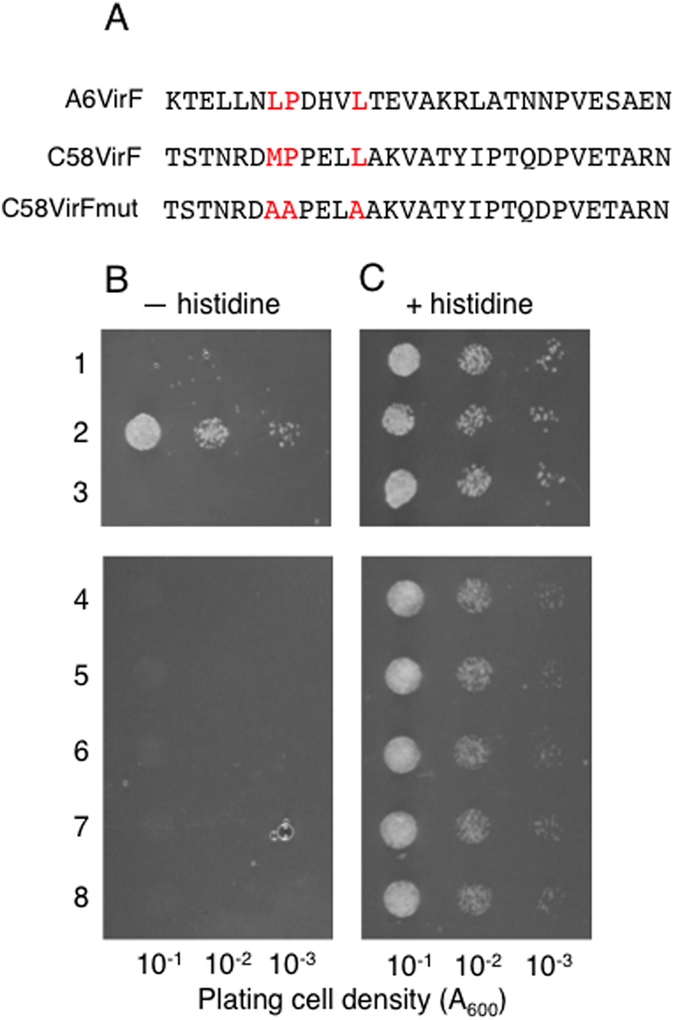
Interaction between C58VirF and ASK depends on the F-box protein domain of C58VirF. (**A**) Amino acid sequences of the F-box protein domain from A6VirF, C58VirF, and C58VirFmut. Substituted conserved residues are highlighted in red. (**B**) Growth in the absence of histidine, tryptophan and leucine. (**C**) Cell growth in the absence of tryptophan and leucine. Lane 1, LexA-C58VirF + Gal4AD; lane 2, LexA-C58VirF + Gal4AD-ASK1; lane 3, LexA-C58VirFmut + Gal4AD-ASK1; lane 4, LexA-C58VirFmut + Gal4AD-ASK1; lane 5, LexA-C58VirFmut + Gal4AD-ASK2; lane 6, LexA-C58VirFmut + Gal4AD; lane 7, LexA-C58VirFmut + Gal4AD-VIP1; lane 8, LexA-C58VirFmut + Gal4AD-VirD2.

Although previous studies indicated that C58VirF lacks apparent biological function^4^,^5^, the homology with octopine-type VirF F-box domain prompted us to investigate the potential functionality of C58VirF as an F-box protein. Specifically, we examined whether the putative F-box domain of C58VirF is biologically functional. To this end, we assayed potential interaction of C58VirF with *Arabidopsis* Skp1-like, ASK, proteins using a yeast-two-hybrid assay, in which protein interaction is indicated by histidine prototrophy^22^ (Figs 3,4). *Arabidopsis* has been reported to contain 21 *ASK* genes in its genome^23^, and we selected nine of their protein products, specifically, ASK1, ASK2, ASK4, ASK5, ASK10, ASK11, ASK13, ASK18, and ASK21, for testing interactions with C58VirF; these proteins were chosen because they represent all seven clades of the phylogenetic tree of this protein family^24^. These experiments detected interaction of C58VirF with ASK1, ASK2, ASK4, ASK13, and ASK18 (Fig. 3A, rows 1, 11, 12, 13, 17, 18), but not with ASK 5, ASK10, ASK11, or ASK21 (Fig. 3A, rows 14, 15, 16, 19). C58VirF also did not interact with any other *Agrobacterium* proteins known to be translocated to host plant cell^6^,^17^,^25^,^26^, i.e., VirE3, VirD2, or VirE2 (Fig. 3A, rows 3, 4, 7). C58VirF also did not interact with VIP1 and VIP2 (Fig. 3A, rows 5, 6), some of the plant proteins thought to be involved in the infection process^27^,^28^,^29^. In positive control experiments, VirE2 interacted with VIP1 (Fig. 3A, row 9) as established previously^28^ whereas, in negative control experiments, no interaction was observed between VirE2 and VirD2 (Fig. 3A, row 8), or between C58VirF or VirE2 preys and the unfused Gal4 activation domain (Gal4AD) encoded by an empty bait vector (Fig. 3A, rows 1, 10). Under the non-selective conditions, all combinations of the tested proteins resulted in the efficient cell growth, indicating that none of the tested constructs interfered with the cell viability (Fig. 3B).

Within the SCF complex, interaction between the F-box protein and its Skp1/ASK partner is mediated by the F-box domain^30^. Thus, we examined whether the F-box domain of C58VirF is required for its interaction with ASK1, the best-studied member of the ASK family^23^. To this end, three point mutations were generated within the ASK1 F-box domain, in which the conserved leucine/methionine, proline, and leucine residues (see Fig. 1A) were substituted with alanines (Fig. 4A). Previously, this type of mutations in the octopine-type VirF were shown to block its interaction with ASK1^11^. Unlike the wild-type C58VirF which bound ASK1 (Fig. 4B, row 2), its F-box domain mutant, designated C58VirFmut, did not interact with ASK1 (Fig. 4B, rows 3, 4) or ASK2 (Fig. 4B, row 5). In negative control experiments, C58VirFmut did not interact with VIP1 or VirD2 (Fig. 4B, rows 7, 8); also neither C58VirF not C58VirFmut interacted with unfused Gal4AD (Fig. 4B, rows 1, 6). Under non-selective conditions, cells in all tested systems remained viable (Fig. 4C).

**Figure 5:**
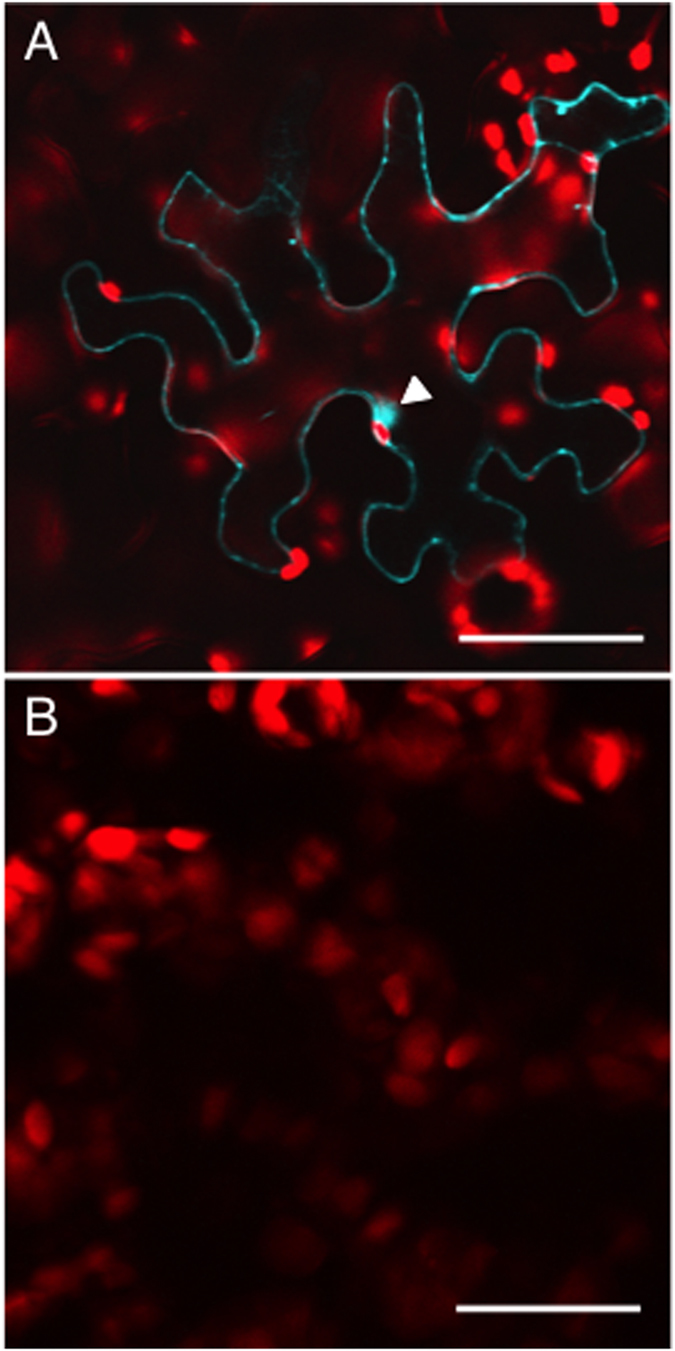
Specific interaction between C58VirF and ASK proteins *in planta*. The indicated combinations of proteins were expressed transiently in *N. benthamiana* leaf mesophyll cells. (**A**) nCerulean-C58VirF + cCFP-ASK1. (**B**) nCerulean-C58VirFmut + cCFP-ASK1. BiFC signal is in blue, plastid autofluorescence is in red. Location of the cell nucleus is indicated by a white arrowhead. Images are single confocal sections. Bars = 30 μm.

Finally, we confirmed the C58VirF-ASK interaction and its dependence on the C58VirF F-box motif directly *in planta*, using bimolecular fluorescence complementation (BiFC). For these verification studies, we chose ASK1 as a representative ASK family member that is recognized by C58VirF (see Fig. 3). C58VirF was tagged with N-terminal fragment of Cerulean fluorescent protein (nCerulean)^31^ whereas ASK1 was tagged with the C-terminal fragment of cyan fluorescent protein (cCFP). Fig. 5A shows that nCerulean-C58VirF and cCFP-ASK1 interacted with each other within living plant cells, producing the BiFC signal. The interacting proteins were located predominantly in the cytoplasm, but also were observed in the cell nucleus. As expected, co-expression of nCerulean-C58VirFmut and cCFP-ASK1 failed to reconstitute the BiFC fluorescence (Fig. 5B); similarly, no signal was detected following co-expression of cCFP-ASK1 and free nCerulean (data not shown).

## Discussion

The current view of the *Agrobacterium* virulence system suggests that the nopaline- and octopine-type T- plasmids encode well-conserved Vir proteins, except for one protein, VirF, which is encoded by the octopine-type, but not by the nopaline-type, Ti plasmid^4^,^5^; in fact, one study explicitly concluded that the *vir*F gene is “absent from the nopaline pTiC58 of *A. tumefaciens*”^14^. On the other hand, the ability of *Agrobacterium* to transform plants genetically depends on the Vir system with each Vir protein playing a role in the transformation process. The lack of conservation of VirF is, therefore, surprising, especially, since VirF represents the only known functional link between the bacterial Vir system and the host UPS^11^,^12^. Thus, we analyzed the area of the nopaline-type *vir* region that corresponds to the octopine-type *vir*F and identified several regions of homology, in particular a sequence which encodes for amino acid residues common for F-box protein domains. This sequence was not detectible *in silico*, but the F-box homology was clearly identified by manual analysis. This F-box domain of C58VirF was biologically active as C58VirF interacted with ASK proteins, an interaction that represents the major functional hallmark of all F-box proteins^15^,^32^,^33^,^34^. Importantly, this interaction was not observed with C58VirF harboring point mutations in the F-box domain, indicating that C58VirF is a *bona fide* F-box protein.

Interestingly, C58VirF interacted with those *Arabidopsis* ASK proteins that belong to the subfamilies expressed at relatively high levels in all type of tissues, while no interaction was detected with ASKs showing a more specific pattern of expression^24^. This interaction specificity of C58VirF was somewhat different from that of the octopine-type VirF, which has been shown to interact with ASK1, ASK2, and ASK10^11^, whereas C58VirF interacted with ASK1 and ASK2, but not with ASK10.

An especially interesting difference between the nopaline-type and the octopine-type VirF proteins was their recognition of VIP1. Our localization studies show that both VirF proteins partition between the cell cytoplasm and the nucleus in plant cells, presumably due to their small size. Furthermore, complexes between the interacting C58VirF and ASK1 proteins, also partitioned between the cytoplasm and the nucleus. In the case of the octopine-type VirF, this localization is compatible with its only known target, VIP1, also shown to partition between the cytoplasm and the nucleus^35^. In the case of infection by the nopaline-type *Agrobacterium*, the host nucleocytoplasmic VBF protein^36^—a functional F-box analog of the octopine-type VirF encoded by the host plant and able to destabilize VIP1 and substitute for the missing VirF in a VirF(-) octopine-type *Agrobacterium* mutant^37^,^38^—may fulfill this function of VIP1 destabilization. Thus, we hypothesize that this difference in VirF targets may correspond to a difference in host specificity between the nopaline and octopine bacterial strains; in this scenario, nopaline-type strains would be less efficient in plant species and/or tissues that do not express an active VBF. That would explain why octopine-type VirF was found to be important for virulence only in some host species, such as *N. glauca*^4^,^5^ and tomato^37^. In contrast, transformation efficiency in maize was lower with an *Agrobacterium* strain expressing octopine VirF, supporting the notion that the effect of VirF on *Agrobacterium* infection varies according to the host plant species and may contribute to the specificity of the host range^39^.

Although the direct targets of C58VirF remain unknown, most likely they represent some of the host cell proteins. Indeed, C58VirF carries a conserved bacterium-to-plant cell export signal and thus functions in the plant cell, either in the cytoplasm or in the nucleus. For example, it is possible that C58VirF targets and destabilizes cellular defense proteins to facilitate the infection further. As additional targets of the octopine- and nopaline-type VirF proteins are discovered, their target specificities may prove to overlap at least in some hosts, especially taking into account that the subcellular patterns of localization for both types of VirF proteins overlap as well.

## Materials and Methods

### Plants

*Nicotiana benthamiana* plants were grown in soil in an environment-controlled growth chamber under long day conditions (16 h light/8 h dark) at 22 °C.

### DNA constructs

For the yeast-two-hybrid experiments, the C58VirF coding sequence—amplified using the primer pair 5′CCGGAATTCATGGAGCCCAGCCAACGAAGC3′/5′CCGCTCGAGTTATCGCGATAGTCCAGAGCGAC3′ and purified Ti plasmid of the wild-type *A. tumefaciens* C58-C1 as template—was inserted into the EcoRI-SalI sites of the LexA plasmid pSTT91 [TRP1^+^ 40,] and Gal4AD plasmid pGAD424 (LEU2^+^, Clontech). LexA fusion of VirE2 in pSTT91, and Gal4AD fusions of VirD2, VirE3, ASK1, VIP1 and VIP2 in pGAD424 were described previously^12^,^25^,^28^,^29^,^41^. For Gal4AD fusions of other ASK proteins, the corresponding cDNAs were amplified using the following primer pairs 5′CCGGAATTCATGTCGACGGTGAGAAAAATC3′/CCGCTCGAGTCATTCAAACGCCCACTGATTC3′ (ASK2), 5′GGAAGATCTGTATGGCAGAAACGAAGAAGATGATC3′/5′CCGCTGCAGTCACTCGAACGCCCACTTGTTC3′ (ASK4), 5′CCGGAATTCATGTCGACGAAGATCATGTTGAAG3′/5′CCGCTCGAGTCATTGAAAAGCCCATTGATTCTC3′ (ASK5), 5′GGAAGATCTGTATGTCGACGAAGAAGATCATATTGAAG3′/5′CCGCTGCAGTCATTCAAAACCCCATTGATTCTCC3′ (ASK10), 5′CCGGAATTCATGTCTTCGAAGATGATCGTGTTG3′/5′CCGCTCGAGTCATTCAAAAGCCCATTGATTCTC3′ (ASK11), 5′CCGGAATTCATGTCGAAGATGGTTATGTTGCTG3′/5′CCGCTCGAGTCATTCAAAAGCCCATTGATTCTCC3′ (ASK13), 5′CCGGAATTCATGGCTTCTTCTTCCGAAGAG3′/5′CCGCTCGAGTTACTCATTAAAAGTCCAAGCATT3′ (ASK18), and 5′CCGGAATTCATGTCAGAAGGTGAAATGGCCATC3′/5′CCGCTCGAGTCACTTGTGTCCTGCAGCTGG3′ (ASK21), and *Arabidopsis* Col0 cDNA library as template, and inserted into EcoRI-SalI (ASK2, ASK5, ASK11, ASK13, ASK18, ASK21) or BglII-PstI sites (ASK4, ASK10) of pGAD424.

For generation of C58VirFmut, coding sequences of the two overlapping N- and C-terminal segments of C58VirF were first amplified with the primer pairs 5′CCGGAATTCATGGAGCCCAGCCAACGAAGC3′/5′GCCGCAAGCTCGGGAGCCGCATCCC3′ and 5′GATGCGGCTCCCGAGCTTGCGGCTAAG3′/5′CCGCTCGAGTTATCGCGATAGTCCAGAGCGAC3′, respectively, introducing the following three mutations: M28A, P29A, and L33A. Then, using these two PCR products as templates, the full coding sequence of C58VirFmut was amplified and cloned into EcoRI-SalI sites of pSTT91 as described above for C58VirF.

For transient expression of the GFP-GUS-C58VirF fusion, the coding sequence of C58virF was amplified using the primer pair 5′CCGGAATTCATGGAGCCCAGCCAACGAAGC3′/5′CCGCTCGAGTTATCGCGATAGTCCAGAGCGAC3′ and digested with EcoRI and SalI, and coding sequence of GUS was amplified using the primer pair 5′GGAAGATCTATGTTACGTCCTGTAGAAACCCC3′/5′CCGGAATTCTTGTTTGCCTCCCTGCTGC3′ and digested with BglII and EcoRI. Both fragments were then inserted by triple ligation into the BglII-SalI sites of pSAT5-MCS^42^. Finally, the coding sequence of the GUS-C58VirF fusion was excised as a BglII-SalI fragment and inserted into the same sites of pSAT1-EGFP-C1^42^. For GFP-C58VirF, the coding sequence of C58VirF was amplified using the primer pair 5′GGAAGATCTATGGAGCCCAGCCAACGAAGC3′/5′CCGCTCGAGTTATCGCGATAGTCCAGAGCGAC3′ and inserted into the BglII-SalI sites of pSAT1-EGFP-C1. For transient expression of the GFP-GFP-VirF fusion, the octopine VirF coding sequence from pVirF (a kind gift from Dr. Stanton Gelvin) was first subcloned into the EcoRI-SmaI sites of pEGFP-C1 (Clontech). Then, into the BglII-HindIII sites of the resulting construct, we inserted an additional copy of the GFP coding sequence, amplified from pEGFP-C1 using the primer pair 5′GGAAGATCTATGGTGAGCAAGGGCG3′/5′CCCAAGCTTGTCCGGACTT GTACAGCTCGTC3′. Finally, the sequence coding for the GFP-GFP-VirF fusion was subcloned into the NcoI-BamHI sites of pRTL2-GUS^43^, replacing GUS. For internal reference of a nucleus-localizing protein, we used RFP-NLS—a fusion between mRFP and NLS of the *Agrobacterium* VirD2 protein^44^—which was expressed from the pSAT6-mRFP-VirD2NLS construct (a kind gift from Dr. Stanton Gelvin).

For BiFC experiments, the coding sequence of ASK1 was amplified with the primer pair 5′AAAGGATTCATGTCTGCGAAGAAGATTGTGTTGAAG3′/5′AAACTGCAGTCATTCAAAAGCCCATTGGTTCTCTC3′ and inserted into the BglII-PstI sites of pSAT4-cCFP-C^31^. The coding sequences of C58VirF and C58VirFmut were amplified using the primer pairs 5′GGAAGATCTATGGAGCCCAGCCAACGAAGC3′/5′CCGCTCGAGTTATCGCGATAGTCCAGAGCGAC3′ and 5′CCGGAATTCTATGGAGCCCAGCCAACGAAGC3′/5′CCGCTCGAGTTATCGCGATAGTCCAGAGCGAC3′ and inserted into the BglII-SalI or EcoRI-SalI sites, respectively, of pSAT6-nCerulean-C^31^. All constructs were verified by DNA sequencing, and all of them expressed proteins from the constitutive tandem 35S RNA promoter of the *Cauliflower mosaic virus* (CaMV)^45^.

### Yeast-two-hybrid protein interaction assay

The assay was performed using the yeast strain L40^22^, co-transformed with pSTT91- and pGAD424-derived plasmids. Five to ten colonies obtained on plates with synthetic defined premixed yeast growth media (TaKaRa Clontech) lacking either leucine and tryptophan (SD-Leu-Trp) or leucine, tryptophan and histidine (SD-Leu-Trp-His) were resuspended in water and plated at different dilutions on the same growth media. Cell growth was recorded after incubation for 2–3 days at 28 °C.

### Transient expression for subcellular localization and BiFC in plant tissues

For biolistic gene delivery, DNA preparations of tested constructs (20 μg of each plasmid) was absorbed onto 10 mg of 1-μm gold particles (Bio-Rad) and microbombarded into *N. benthamiana* leaf epidermis at a pressure of 140–160 psi using a portable Helios gene gun system (Model PDS-1000/He, Bio-Rad), essentially as described^46^ 1. After incubation for 24 h at 22–24 °C, the microbombarded tissues were analyzed under a Zeiss (Oberkochen, Germany) LSM 5 Pascal confocal laser scanning microscope. All experiments were repeated at least three times. For all experiments, a total of at least 15 expressing cells were observed with a similar pattern of subcellular localization of the fluorescence signal.

## Additional Information

**How to cite this article**: Lacroix, B. and Citovsky, V. Nopaline-type Ti plasmid of *Agrobacterium* encodes a VirF-like functional F-box protein. *Sci. Rep.* **5**, 16610; doi: 10.1038/srep16610 (2015).

## References

[b1] Gelvin S. B. *Agrobacterium*-mediated plant transformation: the biology behind the “gene-jockeying” tool. Microbiol. Mol. Biol. Rev. 67, 16–37 (2003). 12626681 10.1128/MMBR.67.1.16-37.2003 PMC150518

[b2] Lacroix B. & Citovsky V. The roles of bacterial and host plant factors in *Agrobacterium*-mediated genetic transformation. Int. J. Dev. Biol. 57, 467–481 (2013). 24166430 10.1387/ijdb.130199bl PMC9478875

[b3] Escobar M. A. & Dandekar A. M. *Agrobacterium tumefaciens* as an agent of disease. Trends Plant Sci. 8, 380–386 (2003). 12927971 10.1016/S1360-1385(03)00162-6

[b4] Melchers L. S. *et al.* Octopine and nopaline strains of *Agrobacterium tumefaciens* differ in virulence; molecular characterization of the *virF* locus. Plant Mol. Biol. 14, 249–259 (1990). 2101693 10.1007/BF00018565

[b5] Regensburg-Tuink A. J. & Hooykaas P. J. J. Transgenic *N. glauca* plants expressing bacterial virulence gene *virF* are converted into hosts for nopaline strains of *A. tumefaciens*. Nature 363, 69–71 (1993). 8479538 10.1038/363069a0

[b6] Vergunst A. C. *et al.* VirB/D4-dependent protein translocation from *Agrobacterium* into plant cells. Science 290, 979–982 (2000). 11062129 10.1126/science.290.5493.979

[b7] Petroski M. D. & Deshaies R. J. Function and regulation of cullin-RING ubiquitin ligases. Nat. Rev. Mol. Cell Biol. 6, 9–20 (2005). 15688063 10.1038/nrm1547

[b8] Lechner E., Achard P., Vansiri A., Potuschak T. & Genschik P. F-box proteins everywhere. Curr. Opin. Plant Biol. 9, 631–638 (2006). 17005440 10.1016/j.pbi.2006.09.003

[b9] Hua Z. & Vierstra R. D. The cullin-RING ubiquitin-protein ligases. Annu. Rev. Plant Biol. 62, 299–334 (2011). 21370976 10.1146/annurev-arplant-042809-112256

[b10] Jin J. *et al.* Systematic analysis and nomenclature of mammalian F-box proteins. Genes Dev. 18, 2573–2580 (2004). 15520277 10.1101/gad.1255304 PMC525538

[b11] Schrammeijer B. *et al.* Interaction of the virulence protein VirF of *Agrobacterium tumefaciens* with plant homologs of the yeast Skp1 protein. Curr. Biol. 11, 258–262 (2001). 11250154 10.1016/s0960-9822(01)00069-0

[b12] Tzfira T., Vaidya M. & Citovsky V. Involvement of targeted proteolysis in plant genetic transformation by *Agrobacterium*. Nature 431, 87–92 (2004). 15343337 10.1038/nature02857

[b13] Magori S. & Citovsky V. Hijacking of the host SCF ubiquitin ligase machinery by plant pathogens. Front. Plant Sci. 2, 87 (2011). 22645554 10.3389/fpls.2011.00087 PMC3355745

[b14] Schrammeijer B., Hemelaar J. & Hooykaas P. J. J. The presence and characterization of a *virF* gene on *Agrobacterium vitis* Ti plasmids. Mol. Plant-Microbe Interact. 11, 429–433 (1998). 9574510 10.1094/MPMI.1998.11.5.429

[b15] Kipreos E. T. & Pagano M. The F-box protein family. Genome Biol. 1, Reviews3002.3001-3002.3007 (2000). 10.1186/gb-2000-1-5-reviews3002 PMC138887 11178263

[b16] Gagne J. M., Downes B. P., Shiu S. H., Durski A. M. & Vierstra R. D. The F-box subunit of the SCF E3 complex is encoded by a diverse superfamily of genes in *Arabidopsis*. Proc. Natl. Acad. Sci. USA 99, 11519–11524 (2002). 12169662 10.1073/pnas.162339999 PMC123288

[b17] Vergunst A. C. *et al.* Positive charge is an important feature of the C-terminal transport signal of the VirB/D4-translocated proteins of *Agrobacterium*. Proc. Natl. Acad. Sci. USA 102, 832–837 (2005). 15644442 10.1073/pnas.0406241102 PMC545537

[b18] Stachel S. E., Messens E., Van Montagu M. & Zambryski P. C. Identification of the signal molecules produced by wounded plant cell that activate T-DNA transfer in *Agrobacterium tumefaciens*. Nature 318, 624–629 (1985).

[b19] Bolton G. W., Nester E. W. & Gordon M. P. Plant phenolic compounds induce expression of the Agrobacterium tumefaciens loci needed for virulence. Science 232, 983–985 (1986). 3085219 10.1126/science.3085219

[b20] Hattori Y. *et al.* Sequence characterization of the *vir* region of a nopaline type Ti plasmid, pTi-SAKURA. Genes Genet. Syst. 76, 121–130 (2001). 11434457 10.1266/ggs.76.121

[b21] Dingwall C. & Laskey R. A. Nuclear targeting sequences - a consensus? Trends Biochem. Sci. 16, 478–481 (1991). 1664152 10.1016/0968-0004(91)90184-w

[b22] Hollenberg S. M., Sternglanz R., Cheng P. F. & Weintraub H. Identification of a new family of tissue-specific basic helix-loop-helix proteins with a two-hybrid system. Mol. Cell. Biol. 15, 3813–3822 (1995). 7791788 10.1128/mcb.15.7.3813 PMC230620

[b23] Kuroda H. *et al.* Classification and expression analysis of *Arabidopsis* F-box-containing protein genes. Plant Cell Physiol. 43, 1073–1085 (2002). 12407186 10.1093/pcp/pcf151

[b24] Dezfulian M. H., Soulliere D. M., Dhaliwal R. K., Sareen M. & Crosby W. L. The *SKP1-like* gene family of *Arabidopsis* exhibits a high degree of differential gene expression and gene product interaction during development. PLOS ONE 7, e50984 (2012). 23226441 10.1371/journal.pone.0050984 PMC3511428

[b25] Lacroix B., Vaidya M., Tzfira T. & Citovsky V. The VirE3 protein of *Agrobacterium* mimics a host cell function required for plant genetic transformation. EMBO J. 24, 428–437 (2005). 15616576 10.1038/sj.emboj.7600524 PMC545813

[b26] Vergunst A. C., van Lier M. C. M., den Dulk-Ras A. & Hooykaas P. J. J. Recognition of the *Agrobacterium* VirE2 translocation signal by the VirB/D4 transport system does not require VirE1. Plant Physiol. 133, 978–988 (2003). 14551327 10.1104/pp.103.029223 PMC281595

[b27] Li J., Krichevsky A., Vaidya M., Tzfira T. & Citovsky V. Uncoupling of the functions of the *Arabidopsis* VIP1 protein in transient and stable plant genetic transformation by *Agrobacterium*. Proc. Natl. Acad. Sci. USA 102, 5733–5738 (2005). 15824315 10.1073/pnas.0404118102 PMC556277

[b28] Tzfira T., Vaidya M. & Citovsky V. VIP1, an *Arabidopsis* protein that interacts with *Agrobacterium* VirE2, is involved in VirE2 nuclear import and *Agrobacterium* infectivity. EMBO J. 20, 3596–3607 (2001). 11432846 10.1093/emboj/20.13.3596 PMC125502

[b29] Anand A. *et al.* *Arabidopsis* VIRE2 INTERACTING PROTEIN2 is required for *Agrobacterium* T-DNA integration in plants. Plant Cell 19, 1695–1708 (2007). 17496122 10.1105/tpc.106.042903 PMC1913729

[b30] Bai C. *et al.* SKP1 connects cell cycle regulators to the ubiquitin proteolysis machinery through a novel motif, the F-box. Cell 86, 263–274 (1996). 8706131 10.1016/s0092-8674(00)80098-7

[b31] Lee L. Y., Fang M. J., Kuang L. Y. & Gelvin S. B. Vectors for multi-color bimolecular fluorescence complementation to investigate protein-protein interactions in living plant cells. Plant Methods 4, 24 (2008). 18922163 10.1186/1746-4811-4-24 PMC2572157

[b32] Xiao W. & Jang J. C. F-box proteins in *Arabidopsis*. Trends Plant Sci. 5, 454–457 (2000). 11077244 10.1016/s1360-1385(00)01769-6

[b33] Eckardt N. A. F-box proteins take center stage. Plant Cell 16, 558–561 (2004).

[b34] Ho M. S., Tsai P. I. & Chien C. T. F-box proteins: the key to protein degradation. J. Biomed. Sci. 13, 181–191 (2006). 16463014 10.1007/s11373-005-9058-2

[b35] Djamei A., Pitzschke A., Nakagami H., Rajh I. & Hirt H. Trojan horse strategy in *Agrobacterium* transformation: abusing MAPK defense signaling. Science 318, 453–456 (2007). 17947581 10.1126/science.1148110

[b36] Zaltsman A., Krichevsky A., Kozlovsky S. V., Yasmin F. & Citovsky V. Plant defense pathways subverted by *Agrobacterium* for genetic transformation. Plant Signal. Behav. 5, 1245–1248 (2010). 20890133 10.4161/psb.5.10.12947 PMC3115358

[b37] Zaltsman A., Krichevsky A., Loyter A. & Citovsky V. *Agrobacterium* induces expression of a plant host F-box protein required for tumorigenicity. Cell Host Microbe 7, 197–209 (2010). 20227663 10.1016/j.chom.2010.02.009 PMC3427693

[b38] García-Cano E., Zaltsman A. & Citovsky V. Assaying proteasomal degradation in a cell-free system in plants. J. Vis. Exp. 85, e51293 (2014). 10.3791/51293 PMC4090386 24747194

[b39] Jarchow E., Grimsley N. H. & Hohn B. *virF*, the host range-determining virulence gene of *Agrobacterium tumefaciens*, affects T-DNA transfer to *Zea mays*. Proc. Natl. Acad. Sci. USA 88, 10426–10430 (1991). 11607242 10.1073/pnas.88.23.10426 PMC52941

[b40] Sutton A. *et al.* A novel form of transcriptional silencing by Sum1-1 requires Hst1 and the origin recognition complex. Mol. Cell. Biol. 21, 3514–3522 (2001). 11313477 10.1128/MCB.21.10.3514-3522.2001 PMC100273

[b41] Tzfira T., Vaidya M. & Citovsky V. Increasing plant susceptibility to *Agrobacterium* infection by overexpression of the *Arabidopsis VIP1* gene. Proc. Natl. Acad. Sci. USA 99, 10435–10440 (2002). 12124400 10.1073/pnas.162304099 PMC124932

[b42] Tzfira T. *et al.* pSAT vectors: a modular series of plasmids for fluorescent protein tagging and expression of multiple genes in plants. Plant Mol. Biol. 57, 503–516 (2005). 15821977 10.1007/s11103-005-0340-5

[b43] Carrington J. C., Freed D. D. & Leinicke A. J. Bipartite signal sequence mediates nuclear translocation of the plant potyviral NIa protein. Plant Cell 3, 953–962 (1991). 1822993 10.1105/tpc.3.9.953 PMC160062

[b44] Howard E., Zupan J., Citovsky V. & Zambryski P. C. The VirD2 protein of *A. tumefaciens* contains a C-terminal bipartite nuclear localization signal: implications for nuclear uptake of DNA in plant cells. Cell 68, 109–118 (1992). 1732061 10.1016/0092-8674(92)90210-4

[b45] Restrepo M. A., Freed D. D. & Carrington J. C. Nuclear transport of plant potyviral proteins. Plant Cell 2, 987–998 (1990). 2136629 10.1105/tpc.2.10.987 PMC159947

[b46] Ueki S., Lacroix B., Krichevsky A., Lazarowitz S. G. & Citovsky V. Functional transient genetic transformation of *Arabidopsis* leaves by biolistic bombardment. Nat. Protoc. 4, 71–77 (2009). 19131958 10.1038/nprot.2008.217

